# Targeting wild-type *TP53* using AMG 232 in combination with MAPK inhibition in Metastatic Melanoma; a phase 1 study

**DOI:** 10.1007/s10637-022-01253-3

**Published:** 2022-05-30

**Authors:** Stergios J. Moschos, Shahneen Sandhu, Karl D. Lewis, Ryan J. Sullivan, Igor Puzanov, Douglas B. Johnson, Haby A. Henary, Hansen Wong, Vijay V. Upreti, Georgina V. Long, Keith T. Flaherty

**Affiliations:** 1 Department of Medicine, Division of Medical Oncology, The University of North Carolina at Chapel Hill and the Lineberger Comprehensive Cancer Center, Chapel Hill, NC USA; 2 Department of Medical Oncology, Peter MaCallum Cancer Center and the University of Melbourne, Melbourne, VIC Australia; 3 Division of Medical Oncology, Anschultz Medical Campus, University of Colorado, Denver, CO USA; 4 Developmental Therapeutics and Melanoma Programs, Massachusetts General Hospital Cancer Center, Boston, MA USA; 5 Department of Medicine, Vanderbilt University Medical Center and Ingram Cancer Center, Nashville TN, USA; 6 Medical Affairs, Amgen Inc, Thousand Oaks, CA USA; 7 Clinical Pharmacology, Modeling & Simulation, Amgen Inc, South San Francisco, CA USA; 8 Melanoma Institute Australia, The University of Sydney and Royal North Shore, and Mater Hospitals, Sydney NSW, Australia

**Keywords:** MDM2 inhibitors, *BRAFV600E*, Dabrafenib, Trametinib, Metastatic melanoma

## Abstract

**Background:**

Targeting the MDM2-p53 interaction using AMG 232 is synergistic with MAPK inhibitors (MAPKi) in preclinical melanoma models. We postulated that AMG 232 plus MAPKi is safe and more effective than MAPKi alone in *TP53*-wild type, MAPKi-naïve metastatic melanoma.

**Methods:**

Patients were treated with increasing (120 mg, 180 mg, 240 mg) oral doses of AMG 232 (seven-days-on, 15-days-off, 21-day cycle) plus dabrafenib (D) and trametinib (T) (Arm 1, *BRAFV600*-mutant) or T alone (Arm 2, *BRAFV600*-wild type). Patients were treated for seven days with AMG 232 alone before adding T±D. Safety and efficacy were assessed using CTCAE v4.0 and RECIST v1.1 criteria, respectively. Pharmacokinetic (PK) analysis was performed at baseline and steady-state levels for AMG 232.

**Results:**

31 patients were enrolled. Ten and 21 patients were enrolled in Arm 1 and Arm 2, respectively. The most common AMG 232-related adverse events (AEs) were nausea (87%), diarrhea (77%), and fatigue (74%). Seven patients (23%) were withdrawn from the study due to AMG 232-related AEs. Three dose-limiting AEs occurred (Arm 1, 180 mg, nausea; Arm 2, 240 mg, grade 3 pulmonary embolism; Arm 2, 180 mg, grade 4 thrombocytopenia). AMG 232 PK exposures were not altered when AMG 232 was combined with T±D. Objective responses were seen in 8/10 (Arm 1) and 3/20 (Arm 2) evaluable patients. The median progression-free survival for Arm 1 and Arm 2 was 19.0 months-not reached and 2.8 months, respectively.

**Conclusion:**

The maximum tolerated dose of AMG 232 for both arms was 120 mg. AMG 232 plus T±D exhibited a favorable PK profile. Although objective responses occurred in both arms, adding AMG 232 to T±D did not confer additional clinical benefit.

**Supplementary information:**

The online version contains supplementary material available at 10.1007/s10637-022-01253-3.

## Introduction

Despite the high rate of somatic mutations in cutaneous melanoma, only a small number of these mutations can be directly pharmacologically targeted. Treatment of *BRAFV600*-mutant metastatic melanoma (MM) with BRAF/MEK inhibitors has been associated with durable responses. However, many patients still die even if treated with immunotherapies [1]. Efforts to concurrently target pathways other than the mitogen-activated protein kinase (MAPK) pathway are usually toxic and have a marginal clinical benefit [2, 3] with the possible exception of programmed cell death protein 1 (PD1)/programmed death-ligand 1(PD-L1) pathway inhibitors [4]. Targeting the second most frequent somatic mutation in melanoma, *NRASQ61*, with single-agent MEK inhibitors was associated with marginal clinical benefit [5]. Therefore, there are no standard treatments specifically for patients with *NRASQ61*-mutant melanoma. MM was under-represented in the National Cancer Institute Molecular Analysis for Therapy Choice (NCI-MATCH). Moreover, of the screened patients, only a quarter was assigned to one of the available treatment arms [6, 7]. Targeting essential proteins that regulate cell death pathways and are intact/non-mutated is an alternative treatment strategy that may increase responses to existing targeted therapies in MM [8].

The *murine double minute-2* (*MDM2*) proto-oncogene encodes a multimeric protein that regulates cellular stress responses [9]. The *MDM2* gene infrequently undergoes genetic aberrations in MM [10, 11]. However, the MDM2 protein is frequently upregulated [11] (or activated) in melanoma secondary to the decreased or absent expression of MDM2 inhibitors, such as p14^ARF^. Among the >100 proteins that the MDM2 protein partners with, its physical interaction with p53 is the most well-studied [12]. Under physiologic conditions, MDM2 prevents p53 from entering the nucleus, inhibits its transactivation domain, and induces p53 ubiquitin-mediated degradation [13]. Various forms of cellular stress can affect interaction among p53 and several other partners, including MDM2, leading to p53 accumulation in the nucleus and activation of cell-cycle arrest, DNA repair, senescence, and programmed cell death programs [13]. In contrast with other cancers, the majority of melanomas express wild-type (WT) *TP53*. [14] Given that both *MDM2* and *TP53* genes are infrequently mutated in melanoma and that inhibition of MDM2 may activate p53’s tumor suppressor program, blocking the p53/MDM2 interaction may be an effective anticancer strategy for *TP53*-WT melanomas. [15]

AMG 232 was developed as a potent and selective piperidinone inhibitor of the MDM2-p53 protein interaction [16]. AMG 232 activates TP53 signaling and inhibits cancer cell proliferation *in vitro*. [16] When combined with inhibitors of either the phosphoinositide 3-kinase (PI3K) or the MAPK signaling pathway, AMG 232 demonstrated synergy in cell death across various *TP53*-WT melanoma cell lines [17]. Daily oral administration of AMG 232 in various *TP53*-WT cancer xenograft models showed dose-dependent antitumor activity with effective doses ranging between 9.1-78 mg/kg [16]. AMG 232 administered at 50 mg/kg dose demonstrated antitumor activity either alone or combined with dabrafenib and trametinib, especially in *TP53*-WT patient-derived melanoma xenografts [8]. This study (ClinicalTrials.gov NCT02110355) assessed the safety, tolerability, pharmacokinetics (PK), and maximum tolerated dose (MTD) of AMG 232 combined with dabrafenib-trametinib or trametinib alone in patients with MM with or without *BRAFV600* mutations, respectively, and without prior treatment with BRAF or MEK inhibitors.

## Patients and methods

### Study design

**Fig. 1:**
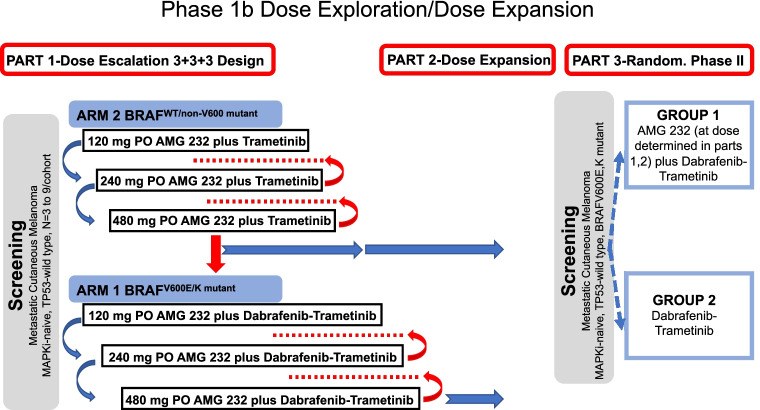
Study design and treatment schema for AMG 232 study in patients with *TP53-WT* MM. Patient enrollment would start from Arm 2 (*BRAFWT*/*non-V600mutant*; AMG 232 in combination with trametinib 2 mg PO QD). AMG 232 (PO QD, 7-days-on, 15-days-off in a 21-day cycle) dose (120 mg, 240 mg, 480 mg) would escalate (blue arrows) until development of dose-limiting toxicity (DLT). Intermediate doses would be investigated and considered between the prespecified dose cohorts only if DLT, or MTD, developed in the immediately higher prespecified dose cohort (red arrows, red dots). The MTD from Arm 2 would be used to drive decisions for AMG 232 dosing in Arm 1 (*BRAFV600*-mutant). Part 2 would expand and refine established MTD dose for AMG 232 across more patients enrolled in each Arm. Part 3 would compare the efficacy of the dabrafenib-trametinib-AMG 232 combination with dabrafenib-trametinib alone in patients with *BRAFV600*-mutant melanoma as part of a randomized (1:1) phase II study. Only Part 1 was completed and therefore presented in this report

Supplementary Information includes major inclusion and exclusion criteria and details regarding the 3-part study design. Figure 1 summarizes the 3-part design. The Institutional Review Board of each participating institution approved the study. All study patients provided written informed consent before enrollment. Each participating institution used an analytically validated molecular test to confirm the non-mutated status of the entire *TP53* gene (*TP53-WT*) and *BRAFV600* mutational status in archival tumor tissues before study enrollment. Patients with a *BRAFV600* mutation were enrolled in Arm 1, and patients with *BRAFV600* wild-type status were enrolled in Arm 2.

We only present data from Part 1 in which AMG 232 was dose-escalated (3+3+3) in Arm 1 and Arm 2. AMG 232 was administered orally at doses of 120, 180, and 240 mg once daily for seven days at every three-week treatment cycle in dose-escalating cohorts. Only for cycle 1, patients received AMG 232 as a single-agent during the first seven days. Trametinib, 2 mg once daily (both Arms), and dabrafenib, 150 mg twice daily (Arm 1 only), were subsequently started on day 8, cycle 1 (Fig. 1). Treatment was administered continuously in consecutive treatment cycles until disease progression, intolerability, or withdrawal of consent.

### Safety and response assessment

Patients were reviewed for safety every three weeks, using the National Cancer Institute Common Terminology Criteria for Adverse Events (NCI-CTCAE) toxicity criteria, v4.0. Supplementary Information includes definitions for the dose-limiting toxicity (DLT) period, non-hematologic DLTs, and other DLT definitions. We decided to establish separate MTDs for each of the treatment arms, because we postulated that they will be different, given that one arm was treated with three drugs (Arm 1, *BRAFV600*-mutant) and the other arm was treated with two drugs (Arm 2, non-*BRAFV600*-mutant). A patient was considered DLT-evaluable if he/she experienced a DLT or otherwise received ≥85% of AMG 232 over 28 days following the first dose. In the event of a DLT or other clinically significant AEs in any part of the study, treatment was withheld, and supportive therapy was administered as clinically indicated. If toxicity resolved to baseline or grade 1 in ≤14 days of stopping therapy, treatment could be restarted. Alternatively, if toxicity was attributed to AMG 232 and did not resolve in 14 days despite symptomatic management (e.g., antiemetics for nausea and vomiting), withdrawal from AMG 232 was recommended. Either trametinib and/or dabrafenib were dose-reduced if toxicity appeared related to any of the two drugs. A dose reduction below 75 mg twice daily for dabrafenib and 1 mg once daily for trametinib was not allowed. If trametinib was dose-reduced to <1 mg once daily, trametinib was permanently discontinued. In that case, however, the patient was allowed to continue dabrafenib and AMG 232. AMG 232 could be discontinued at the patient’s request or safety concerns. Response assessment by the Response Evaluation Criteria in Solid Tumors (RECIST) v1.1 was performed by local review four weeks and eight weeks after cycle 1, day 1, then every eight weeks after that using contrast-enhanced computerized tomography or magnetic resonance imaging of the chest, abdomen, and pelvis.

### Pharmacokinetic assessments

We collected PK samples in cycle 1, day 7 (end of single-agent AMG 232 run-in phase; predose, 30 min, 1 hour, 2 hours, 4 hours, and 6 hours), day 8 (predose), and day 21 (predose, 30 min, 1, hours, 2 hours, 4 hours, and 6 hours), and in cycle 2, day 1 (predose), day 7 (predose, 30 min, 1 hour, 2 hours, 4 hours, and 6 hours), and day 8 (predose). Based on AMG 232 half-life estimates reported previously [18], AMG 232 drug levels were deemed steady-state on cycle 1, day 7, and cycle 2, day 7. Supplementary Information provides details about determining plasma concentrations and other PK parameter estimates for AMG 232, the AMG 232 glucuronide, trametinib, dabrafenib, and hydroxy- and desmethyl-dabrafenib metabolites.

### Statistical analysis

We included patients who received ≥1 dose of AMG 232 and had ≥1 post-baseline efficacy assessment in the efficacy endpoint analysis. In addition, patients who received ≥1 dose of AMG 232 and completed 28 days after the initial AMG 232 dose or discontinued due to toxicity were evaluable for DLT. The sample size for part 1 was based on the 3+3+3 design [20]. Descriptive statistics were provided for demographics, treatment-emergent AEs, and efficacy and included means, medians, standard deviations, and ranges. In addition, we performed exploratory survival analyses using the Kaplan-Meier method for each Arm.

## Results

### Patients

Thirty-one patients (Arm 1, n=10; Arm 2, n=21) with BRAF and MEK inhibitor-naïve MM were enrolled in part 1 of the study between December 2014 and May 2017 across six academic institutions in the United States of America and Australia. Table 1 shows patient baseline characterstics. Most patients had cutaneous melanoma (n=19, 61%), were <65 years old (n=24, 77%), had an Eastern Cooperative Oncology Group (ECOG) performance status of 0 (61%), and had received prior anticancer therapies (n=25, 81%). Of the previously treated patients, the majority (n=22, 88%) had received ≥2 systemic therapies, particularly PD1/PDL-1 inhibitor-based (n=21, 84%).

**Table 1:** Baseline Demographics – Part 1 (n=31)

**Age** (yrs; median, range)	**57 (24,79)**
18-64	24 (77)
≥65	7 (23)
**Sex (%)**	
Male	16 (52)
Female	15 (48)
**Melanoma Subtypes (%)**	
Cutaneous	19 (61)
Acral	5 (16)
Mucosal	2 (6)
Unknown Primary	5 (16)
**AJCC staging (v7) at Original Diagnosis**	
IIIB	1 (3)
IIIC	4 (13)
M1a	8 (26)
M1b	3 (10)
M1c	15 (48)
***BRAFV600* mutation status**	
Yes (Group 1, all *BRAFV600E*)	10 (32)
No (Group 2)	21 (68)
**Prior Cancer Therapies (number of patients, %)**	
No	6 (19)
Yes	25 (81)
Received PD1/PD-L1i only	2
Received ipilimumab only	2
Received PD1/PD-L1i + ipilimumab	19
Did not receive PD1/PD-L1i or ipilimumab	2
**Cancer Treatment types (total)**	**73**
PD1/PD-L1i	27 (37)
Ipilimumab	21 (29)
Non-PD1/PD-L1/CTLA4i	2 (3)
HDIL-2	6 (8)
High-dose interferon-α2b	2 (3)
Targeted therapies	2 (3)
Chemotherapy alone	7 (10)
Intralesional treatment	3 (4)
Other	3 (4)
**Number of Patients with x Lines of Prior Therapy**	
1 prior treatment	3 (10)
2 prior treatments	8 (26)
3 prior treatments	7 (23)
4 prior treatments	5 (16)
≥5 prior treatments	2 (6)
**ECOG Performance Status at Screening**	
0	19 (61)
1	10 (32)
2	2 (6)

*PD1/PD-L1i, PD1/PD-L1* inhibitors (pembrolizumab, nivolumab, avelumab), *AJCC* American Joint Committee on Cancer, *HDIL-2* high-dose bolus interleukin-2, *IC* immune checkpoint proteins

All patients received ≥1 dose of AMG 232; 22 (71%) patients were discontinued from study due to: death from progressive disease (n=17, 55%), decision by sponsor (n=1, 3%), protocol-specified criteria (n=2, 6.5%), and withdrawal of study consent (n=2, 6.5%). All had been discontinued from AMG 232 at the time of the final analysis. Specifically, the reasons for discontinuing AMG 232 were progressive disease (n=15, 48%), AEs (n=7, 23%), withdrawal by patient (n=7, 23%), death (n=1, 3%), and decision by sponsor (n=1, 3%). Reasons for discontinuing trametinib were progressive disease (n=14, 45%), AEs (n=6, 19%), withdrawal by patient (n=7, 23%), death (n=2, 65%), decision by sponsor (n=1, 3%), and non-compliance (n=1, 3%). Reasons for discontinuing dabrafenib for the 10 patients enrolled in Arm 1 were progressive disease (n=3, 33%), AEs (n=2, 20%), and withdrawal by patient (n=5, 50%).

### Dose escalation

In Arm 2, the first arm where study accrual initiated, the first two AMG 232 doses evaluated in combination with trametinib were 120 mg (n=6) and 240 mg (n=6). While no DLTs occurred in the 120 mg dose cohort, a patient treated with 240 mg developed a grade 3 pulmonary embolism on day 13 from initiation of AMG 232. This AE was attributed to both AMG 232 and trametinib and was considered a DLT. We therefore treated 9 patients with the intermediate dose (180 mg). However, a patient treated with the intermediate AMG 232 dose developed grade 4 thrombocytopenia on day 27, after the first cycle. This AE was attributed to AMG 232, was resolved on day 55, and was considered a DLT. Based on the two DLTs, one in each of the 180 mg and 240 mg dose cohorts, as well as the high frequency of gastrointestinal toxicities causally attributed to AMG 232 (Table 2), the sponsor’s study team leadership and site clinical investigators determined that the MTD of the seven-days-on, 14-days-off AMG 232 schedule combined with trametinib was 120 mg once daily.

**Table 2:** Summary of the most frequent treatment-related AEs. ‘Any drug’ implies AEs attributed to either dabrafenib (dabraf), trametinib (tram), or AMG 232

	**Group 1 (BRAF**^**V600**^^**E/K**^**)**				**Group 2 (BRAF**^***WT* OR non-*V600***^**)**						**All Patients**	
	**AMG 232 + Trametinib + Dabrafenib**				**AMG 232 + Trametinib**							
	120 mg		180 mg		120 mg		180 mg		240 mg			
	N=4 (%)		N=6 (%)		N=6 (%)		N=9 (%)		N=6 (%)		**N=31 (%)**	
**Summary of AEs**												
Grade 1	4 (100)		6 (100)		6 (100)		9 (100)		6 (100)		**31 (100)**	
Grade 2	4 (100)		6 (100)		6 (100)		8 (89)		6 (100)		**30 (97)**	
Grade 3	2 (50)		4 (67)		6 (100)		7 (78)		6 (100)		**25 (81)**	
Grade 4	0		0		1 (17)		1 (11)		0		**2 (6)**	
Leading to AMG 232 d/c	1 (25)		1 (17)		1 (17)		2 (22)		2 (33)		**7 (23)**	
Leading to tram d/c	1 (25)		1 (17)		1 (17)		1 (11)		2 (33)		**6 (19)**	
Leading to dabraf d/c	1 (25)		1 (17)		N/A						**2 (6)**	
**Most frequent (>20% patients) AEs according to drug type**												
	Attributed to … (%)		Attributed to ... (%)		Attributed to ... (%)		Attributed to ... (%)		Attributed to … (%)		Attributed to … (%)	
	Any drug	AMG 232 only	Any drug	AMG 232 only	Any drug	AMG 232 only	Any drug	AMG 232 only	Any drug	AMG 232 only	Any drug	AMG 232 only
Nausea	4 (100)		6 (100)		6 (100)		6 (67)	5 (56)	6 (100)		**28 (90)**	**27 (87)**
Diarrhea	4 (100)		6 (100)		5 (83)	4 (67)	6 (67)	5 (56)	6 (100)	5 (83)	**27 (87)**	**24 (77)**
Fatigue	4 (100)		4 (67)		5 (83)		8 (89)	6 (67)	4 (67)		**25 (81)**	**23 (74)**
Vomiting	3 (75)		5 (83)		6 (100)		6 (67)	5 (56)	2 (33)		**22 (71)**	**21 (68)**
Decreased appetite	2 (50)	1 (25)	5 (83)	4 (67)	3 (50)		3 (33)	2 (22)	3 (50)		**16 (52)**	**13 (42)**
Headache	2 (50)	0	4 (67)	1 (17)	4 (67)	2 (33)	2 (22)	0	3 (50)	0	**15 (48)**	**3 (10)**
Dermatitis acneiform	1 (25)		1 (17)	0	3 (50)	0	5 (56)	1 (11)	4 (67)	1 (17)	**14 (45)**	**3 (10)**
Peripheral Edema	1 (25)	0	2 (33)	0	4 (67)	1 (17)	3 (33)	0	3 (50)	0	**13 (42)**	**1 (3)**
Pruritus	3 (75)	0	2 (33)	0	2 (33)	0	2 (22)	0	2 (33)	0	**11 (35)**	**0**
Pyrexia	2 (50)	0	4 (67)	1 (17)	3 (50)	1 (17)	1 (11)	0	1 (17)	0	**11 (35)**	**2 (6)**
Anemia	1 (25)	0	1 (17)		3 (50)	2 (33)	1 (11)	0	3 (50)	2 (33)	**9 (29)**	**5 (16)**
Dehydration	1 (25)	0	2 (33)	0	3 (50)	1 (17)	2 (22)	0	1 (17)	0	**9 (29)**	**1 (3)**
Hypotension	2 (50)	0	2 (33)	1 (17)	0		2 (22)	0	2 (33)	0	**8 (26)**	**1 (3)**
Cough	1 (25)	0	2 (33)	0	2 (33)		1 (11)	0	2 (33)	0	**8 (26)**	**2 (6)**
Chills	1 (25)	0	3 (50)	2 (33)	1 (17)	0	2 (22)	0	1 (17)	0	**8 (26)**	**2 (6)**
Hypertension	2 (50)	0	3 (50)	2 (33)	2 (33)	0	1 (11)	0	0		**8 (26)**	**2 (6)**
Thrombocytopenia	0		1 (17)	0	2 (33)		1 (11)	0	3 (50)	1 (17)	**7 (23)**	**3 (10)**
Rash	1 (25)	0	1 (17)		1 (17)	0	3 (33)	1 (11)	1 (17)	0	**7 (23)**	**2 (6)**
Abdominal pain	1 (25)		1 (17)	0	0		2 (22)		2 (33)		**6 (19)**	**5 (16)**
Dysgeusia	0		2 (33)	0	1 (17)		1 (11)		2 (33)		**6 (19)**	**4 (13)**
AST increased	1 (25)	0	3 (50)	2 (33)	1 (17)		1 (11)	0	0		**6 (19)**	**3 (10)**
Dry mouth	2 (50)	0	1 (17)		1 (17)		0		1 (17)		**5 (16)**	**3 (10)**
Neutropenia	0		2 (33)	1 (17)	2 (33)	1 (17)	0		1 (17)		**5 (16)**	**3 (10)**
Dizziness	1 (25)		2 (33)	1 (17)	1 (17)	0	0		1 (17)		**5 (16)**	**3 (10)**
Pulmonary embolism	0		0		2 (33)	0	1 (11)	0	2 (33)		**5 (16)**	**2 (6)**
Lipase increased	0		3 (50)	2 (33)	0		1 (11)		1 (17)		**5 (16)**	**4 (13)**
Malaise	1 (25)		1 (17)		0		0		2 (33)		**4 (13)**	**4 (13)**
Dyspepsia	1 (25)		2 (33)	1 (17)	0		0		1 (17)		**4 (13)**	**3 (10)**
Seizure	0		1 (17)	0	0		1 (11)	0	1 (17)	0	**3 (10)**	**0**
Adrenal insufficiency	0		0		0		1 (11)	0	1 (17)	0	**2 (6)**	**0**
Cellulitis	0		0		1 (17)	0	1 (11)	0	0		**2 (6)**	**0**
**Serious AEs**												
Nausea	0		1 (17)		2 (33)		1 (11)		1 (17)		**5 (16)**	
Vomiting	1 (25)		1 (17)		2 (33)		1 (11)		0		**5 (16)**	
Pyrexia	1 (25)		2 (33)		1 (17)		1 (11)		0		**5 (16)**	
Pulmonary embolism	0		0		2 (33)		1 (11)		2 (33)		**5 (16)**	
Diarrhea	1 (25)		0		1 (17)		0		2 (33)		**4 (13)**	
Dehydration	0		0		2 (33)		1 (11)		0		**3 (10)**	
Seizure	0		1 (17)		0		1 (11)		1 (17)		**3 (10)**	
Adrenal insufficiency	0		0		0		1 (11)		1 (17)		**2 (6)**	
Cellulitis	0		0		1 (17)		1 (11)		0		**2 (6)**	
Fatigue	0		0		1 (17)		0		0		**1 (3)**	
Peripheral Edema	0		1 (17)		0		0		0		**1 (3)**	
Anemia	0		0		0		0		1 (17)		**1 (3)**	
Hypotension	0		0		0		1 (11)		0		**1 (3)**	
Thrombocytopenia	0		0		0		1 (11)		0		**1 (3)**	

d/c, discontinue; AE, adverse events; N/A, non applicable; %, percentage of patients experiencing adverse event

Based on the experience from Arm 2, the AMG 232 doses evaluated in Arm 1 were 120 mg (n=4) and 180 mg (n=6) combined with trametinib and dabrafenib. There were no DLTs for the 120 mg dose cohort. However, one patient treated with 180 mg AMG 232 had <75% of AMG 232 during the DLT period due to protracted AMG 232-related grade 3 nausea that begun on day 11 and was considered a DLT. Based on the single DLT in the 180 mg dose cohort as well as the high frequency of gastrointestinal toxicities causally attributed to AMG 232 (Table 2), the sponsor’s study team leadership and site clinical investigators determined that the MTD of the seven-days-on, 14-days-off AMG 232 schedule combined with dabrafenib plus trametinib was 120 mg once daily.

Following accrual in the 180 mg dose cohort in Arm 1, the study was completed because the sponsor decided to cease further clinical development of AMG 232.

### Safety and tolerability

A total of 614 treatment-emergent AEs occurred (Table 2). The most common (occurring in ≥20% of patients) AEs were nausea (90%), diarrhea (87%), fatigue (81%), vomiting (71%), decreased appetite (52%), headache (48%), acneiform dermatitis (45%), peripheral edema (42%), pruritus and pyrexia (35.5% each). 83 (13.5%) of the treatment-emergent AEs were considered serious (SAEs) and occurred in 24 (77%) patients. These were predominantly gastrointestinal symptoms, including nausea (16%), vomiting (16%), and diarrhea (13%). Diarrhea could be managed with dietary modifications (discontinue lactose-containing products, BRAT diet, and small-meal portions), hydration, and loperamide (up to a maximum daily dose of 16 mg). Nausea was managed with aprepitant, dexamethasone, and compazine. Table 2 shows a summary of the most frequent (≥20% of patients) treatment-emergent AEs and SAEs according to dose level and Arm.

240 treatment-emergent AEs (39%) were considered by the investigators to be attributable to AMG 232 and occurred in 30 subjects during the study. The most common AMG 232-related AEs (occurring in ≥10% of subjects) were nausea (87%), diarrhea (77%), fatigue (74%), vomiting (68%), decreased appetite (42%), thrombocytopenia (23%), abdominal pain and anemia (16% each); dysgeusia, increased lipase, and malaise (13% each). Most AMG 232-related AEs (26%) were of grade 1 or grade 2. Serious AMG 232-related AEs were reported in 8 patients; nausea (13%), diarrhea (10%), vomiting (10%), and pulmonary embolism (6.5%) were the most frequent. Withdrawals from AMG 232 due to AEs were reported for seven subjects (23%), such as grade 3 arterial injury; grade 2 and 3 nausea; grade 3 diarrhea, nausea and vomiting; grade 1 thrombocytopenia; grade 2 anemia and acneiform dermatitis; grade 5 (death from MM); grade 1 diarrhea; and grade 2 fatigue. Two patients in Arm 1 who had complete antitumor response stopped treatment due to preference; one patient did not tolerate AMG 232 due to gastrointestinal side effects (6-1012) and the other subject stopped all three drugs due to high frequency of doctor’s visits for the trial (6-1011).

### Phamacokinetics

**Fig. 2:**
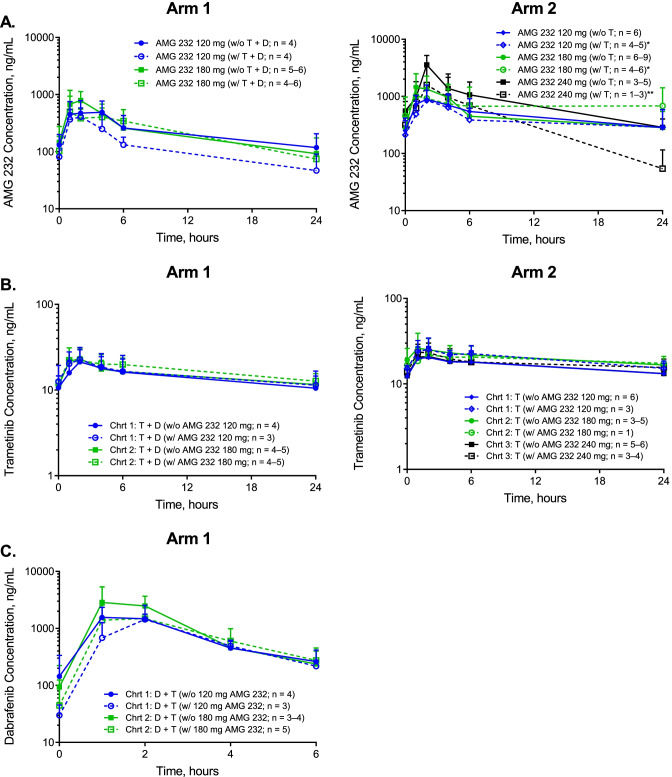
Plasma pharmacokinetics of AMG 232, trametinib, and dabrafenib at steady-state for combinations of AMG 232 with trametinib and dabrafenib (Arm 1) or trametinib (Arm 2) given orally in adult subjects with MM. In Arms 1 and 2, AMG 232 was administered QD on days 1–7 of each cycle with a 14-day treatment-free period on days 8–21. In addition, trametinib was dosed continuously QD starting on day 8 of cycle 1. In Arm 1 only, dabrafenib was dosed continuously BID starting on day 8 of cycle 1. A. Mean (+SD) plasma concentration-time profiles of AMG 232 following AMG 232 dosing on cycle 1, day 7, when administered alone or on cycle 2, day 7, when co-administered with trametinib and dabrafenib [Arm 1] or trametinib [Arm 2]. B. Mean (+SD) plasma concentration-time profiles of trametinib following dosing of trametinib and dabrafenib [Arm 1] or trametinib alone [Arm 2] on cycle 1, day 21, without co-administration of AMG 232 (at the end of the 14-day AMG 232 treatment-free period) or on cycle 2, day 7, when co-administered with AMG 232. C. Mean (+SD) plasma concentration-time profiles of dabrafenib following dosing of dabrafenib and trametinib in Arm 1 on cycle 1, day 21, without co-administration of AMG 232 (at the end of the 14-day AMG 232 treatment-free period) or on cycle 2, day 7, when co-administered with AMG 232. *Abbreviations*: BID, twice a day; Chrt, cohort; D, dabrafenib; QD, once daily; SD, standard deviation; T, trametinib

Figure 2a shows plasma PK profiles of AMG 232 at steady-state for both arms. Table 3 shows PK parameter estimates of AMG 232**.** As previously shown [19], following dosing on cycle 1, day 7, the last of a 7-day treatment of AMG 232 daily monotherapy, AMG 232 was absorbed rapidly (median t_max_ of 2.0 hours) across dose cohorts in both arms. In addition, AMG 232 plasma exposures, as assessed by C_max_ and AUC_24h_, generally increased with increasing AMG 232 dose in both arms (doses from 120 to 180 mg in Arm 1 and 120 to 240 mg in Arm 2 dose ranges in Arms 1 and 2, respectively). In addition, in Arm 1, we observed 1.7- and 1.1-fold increases in the geometric mean C_max_ and AUC_24hr_, respectively, for a 1.5-fold increase in dose. In Arm 2, we observed a 3.6- and 2.2-fold increase in the geometric mean C_max_ and AUC_24hr_ for a 2-fold increase in dose. Finally, mean terminal half-life (t_1/2,z_) and geometric mean apparent clearance (CL/F) across dose cohorts in both arms were 7.04 to 8.32 hours and 11.4 to 30.6 L/hour, respectively.

**Table 3:** Descriptive statistics of AMG 232 plasma PK parameter estimates after oral administration of AMG 232 for 6 days at steady-state levels on cycle 1, day 7, without trametinib (and dabrafenib for Arm 1) and cycle 2, day 7, with trametinib (and dabrafenib for Arm 1) in subjects with metastatic melanoma

**Arm**	**Cohort**	**AMG 232 dose (mg)**	**t**_**max**_** (hr)**	**C**_**max**_** (ng/mL)**	**AUC**_**24hr**_ **(hr•ng/mL)**	**t**_**1/2,z**_ **(hr)**	**CL/F** **(L/hr)**
Cycle 1, Day 7 (AMG 232 monotherapy; prior to co-administration with T + D [Arm 1] or T alone [Arm 2)							
Arm 1: AMG 232 + T + D	1	120	2.0 (1.0–4.0) n = 4	491 (59.4%) n = 4	5140 (59.4%) n = 4	7.04 (NR) n = 1	23.4 (53.6%) n = 4
	2	180	2.0 (1.0–4.0) n = 6	847 (51.3%) n = 6	5890 (34.6%) n = 5	7.79 (0.718) n = 4	30.6 (37.5%) n = 5
Arm 2: AMG 232 + T	1	120	2.0 (2.0–4.0) n = 6	914 (37.4%) n = 6	9810 (61.4%) n = 6	8.32 (NR) n = 2	12.2 (69.7%) n = 6
	2	180	2.0 (0.0–4.0) n = 7	1250 (62.2%) n = 7	10600 (71.7%) n = 7	7.54 (NR) n = 2	16.9 (115.6%) n = 7
	3	240	2.0 (2.0–2.0) n = 4	3300 (46.5%) n = 4	21100 (28.3%) n = 3	8.12 n = 1	11.4 (24.7%) n = 3
Cycle 2, Day 7 (AMG 232 with co-administration of T + D [Arm 1] or T alone [Arm 2])							
Arm 1: AMG 232 + T + D	1	120	2.0 (2.0–4.0) n = 4	392 (43.3%)^a^ n = 4	3140 (31.4%)^a^ n = 4	5.86 (NR) n = 2	38.2 (32.5%) n = 4
	2	180	3.0 (1.0–6.0) n = 6	647 (44.3%)^a^ n = 6	4750 (38.7%)^a^ n = 4	6.93 (NR) n = 2	37.9 (38.2%) n = 4
Arm 2: AMG 232 + T	1	120	3.0 (2.0–6.0) n = 4	630 (77.2%)^a^ n = 4	6740 (62.9%)^a^ n = 4	ND n = 0	17.8 (101.2%) n = 4
	2	180	1.0 (1.0–6.0) n = 5	1010 (52.0%)^a^ n = 5	9410 (107.0%)^a^ n = 5	ND n = 0	19.1 (99.6%) n = 5
	3	240	1.5 (1.0–2.0) n = 2	953 (NR)^b^ n = 2	6280 (NR)^b^ n = 2	5.30 (NR) n = 2	38.2 (NR) n = 2

Data are presented as geometric mean (CV%) except for t_max_ and t_1/2,z_, which were presented as median (minimum-maximum) and mean (SD), respectively. Number of subjects (n) are presented for each parameter

*AUC*_*24h*_ area under the concentration-time curve from time 0 to 24 hrs post-dose, *CL/F* apparent drug clearance after extravascular administration, *C*_*max*_ maximum observed plasma concentration, *CV%* percent coefficient of variation, *D* dabrafenib (150 mg twice daily dosing), *hr* hour, *ND* no data, *NR* not reported, *SD* standard deviation, *T* *trametinib* (2 mg once daily dosing), *t*_*1/2,z*_ terminal half-life, *t*_*max*_ time to reach C_max_

^a^ p>0.05 comparing AMG 232 C_max_ and AUC_24h_ values for subjects following cycle 1, day 7 (AMG 232 monotherapy), and cycle 2, day 7 (AMG 232 in combination with trametinib and dabrafenib or trametinib alone, Wilcoxon matched-pairs signed῏rank test)

^b^ Wilcoxon test not performed (insufficient subjects to conduct test)

**Table 4:** Descriptive statistics of trametinib plasma pharmacokinetic parameter estimates after oral administration of trametinib (and dabrafenib for Arm 1) at steady-state levels on cycle 1, day 21, without AMG 232 and cycle 2, day 7, with AMG 232 in adult subjects with metastatic melanoma

**Arm**	**Cohort and AMG 232 dose given QD on days 1–7 of each cycle**	**Cycle 1, Day 21** **(without AMG 232 co-adminstered)**				**Cycle 2, Day 7** **(with AMG 232 co-administered)**		
		**t**_**max**_** (hr)**	**C**_**max**_** (ng/mL)**	**AUC**_**24hr**_ **(hr•ng/mL)**		**t**_**max**_** (hr)**	**C**_**max**_** (ng/mL)**	**AUC**_**24hr**_ **(hr•ng/mL)**
Arm 1: AMG 232 + T + D	Cohort 1: 120 mg	2.0 (2.0–2.0) n = 4	20.1 (38.7%) n = 4	326 (38.6%) n = 4		2.0 (1.0–2.0) n = 3	22.4 (36.9%)^a^ n = 3	338 (47.3%)^a^ n = 3
	Cohort 2: 180 mg	2.0 (1.0–2.0) n = 5	24.1 (23.2%) n = 5	362 (17.2%) n = 5		2.0 (1.0–6.0) n = 5	25.9 (22.3%)^a^ n = 5	402 (22.5%)^a^ n = 4
Arm 2: AMG 232 + T	Cohort 1: 120 mg	1.5 (0.0–4.0) n = 6	21.5 (33.5%) n = 6	370 (30.5%) n = 6		2.0 (2.0–4.0) n = 3	24.8 (33.7%)^a^ n = 3	468 (23.5%)^a^ n = 3
	Cohort 2: 180 mg	4.0 (1.0–6.0) n = 5	25.3 (31.1%) n = 5	471 (26.7%) n = 5		2.0 n = 1	21.8^b^ n = 1	463^b^ n = 1
	Cohort 3: 240 mg	2.0 (1.0–4.0) n = 6	22.1 (25.0%) n = 6	385 (22.9%) n = 6		2.0 (1.0–4.0) n = 4	24.3 (22.5%)^a^ n = 4	351 (41.9%)^a^ n = 4

Data are presented as geometric mean (CV%) except for t_max_, which was presented as median (minimum-maximum). In addition, number of subjects (n) are presented for each parameter

*AUC*_*24h*_ area under the concentration-time curve from time 0 to 24 hrs post-dose, *C*_*max*_ maximum observed plasma concentration, *CV%* percent coefficient of variation, *D* dabrafenib (150 mg twice daily dosing), *hr* hour, *QD* once daily, *T* trametinib (2 mg QD), *t*_*max*_ time to reach C_max_.

^a^ p > 0.05 comparing trametinib C_max_ and AUC_24h_ values for subjects following cycle 1 day 21 (without AMG 232) and cycle 2 day 7 (with AMG 232) (Wilcoxon matched-pairs signed῏rank test)

^b^ Wilcoxon test not performed (insufficient subjects to conduct test)

**Table 5:** Descriptive statistics of dabrafenib plasma pharmacokinetic parameter estimates after oral administration of trametinib (and dabrafenib for Arm 1) at steady-state levels on cycle 1, day 21, without AMG 232 and cycle 2, day 7, with AMG 232 in adult subjects with metastatic melanoma

**Arm**	**Cohort and AMG 232 dose given QD on days 1–7 of each cycle**	**Cycle 1, Day 21** **(T + D without AMG 232 co-adminstered)**				**Cycle 2 Day 7** **(T + D with AMG 232 co-administered)**		
		**t**_**max**_** (hr)**	**C**_**max**_** (ng/mL)**	**AUC**_**6hr**_ **(hr•ng/mL)**		**t**_**max**_** (hr)**	**C**_**max**_** (ng/mL)**	**AUC**_**6hr**_ **(hr•ng/mL)**
Arm 1: AMG 232 + T + D	Cohort 1: 120 mg	1.0 (1.0–2.0) n = 4	1740 (20.9%) n = 4	4840 (28.0%) n = 4		2.0 (2.0–2.0) n = 3	1200 (76.3%)^a^ n = 3	3640 (60.5%)^a^ n = 3
	Cohort 2: 180 mg	1.0 (1.0–2.0) n = 4	3310 (52.2%) n = 4	7450 (46.8%) n = 4		2.0 (1.0–2.0) n = 5	1750 (40.5%)^a^ n = 5	4910 (30.0%)^a^ n = 5

Data are presented as geometric mean (CV%) except for t_max_, which was presented as median (minimum-maximum). In addition, number of subjects (n) are presented for each parameter

*AUC*_*6h*_ area under the concentration-time curve from time 0 to 6 hrs post-dose, *C*_*max*_ maximum observed plasma concentration, *CV%* percent coefficient of variation, *D* dabrafenib (150 mg twice daily dosing), *hr* hour, *ND* no data, *NR* not reported, *QD* once daily, *T* trametinib (2 mg QD), *t*_*max*_ time to reach C_max_

^a^ p > 0.05 comparing dabrafenib C_max_ and AUC_24h_ values for subjects following cycle 1 day 21 (without AMG 232) and cycle 2 day 7 (with AMG 232) (Wilcoxon matched-pairs signed-rank test)

AMG 232 absorption remained rapid (median t_max_ of 1.0–3.0 hours) when AMG 232 was combined with dabrafenib plus trametinib or trametinib alone. When combined with trametinib and dabrafenib or trametinib alone, exposure to AMG 232 (C_max_ and AUC_24hr_), increased dose-proportionally over the dose range of 120 to 180 mg. In Arm 2, increases in the geometric mean C_max_ and AUC values were not observed between dose levels of 180 and 240 mg, although there were limited data available with only two patients treated at the 240 mg dose level.

Supplementary Information, Fig. 2b and c, Tables 4 and 5 summarize plasma PK profiles of AMG 232, trametinib (alone and in combination with dabrafenib), and dabrafenib at steady-state with and without co-administration of AMG 232. In summary, AMG 232 PK exposures were not significantly altered when AMG 232 was combined with trametinib, with and without dabrafenib. In addition, PK parameter estimates for both trametinib and dabrafenib were within range of those observed in previous studies following repeat dosing monotherapy for each drug.

### Antitumor activity

**Fig. 3:**
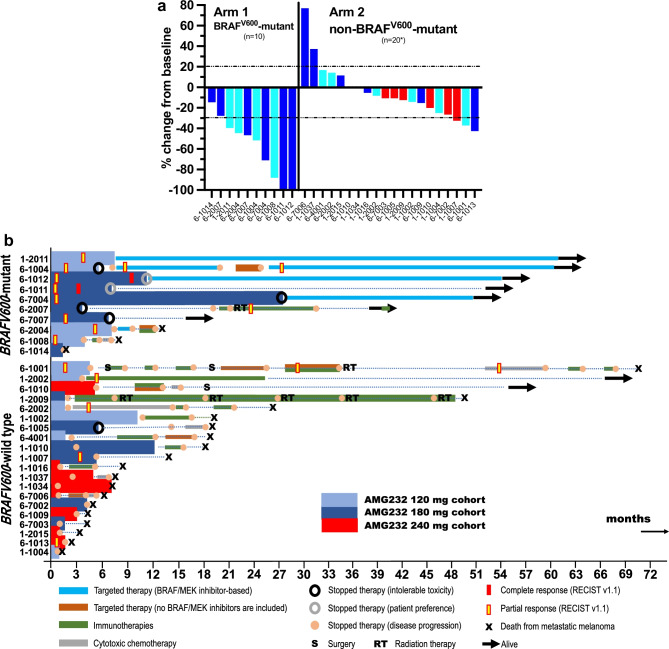
Antitumor benefit of AMG 232 in combination with trametinib alone or trametinib plus dabrafenib in patients with unresectable stage III/IV melanoma (*evaluable patients n=30, see text for details). A. Waterfall plots corresponding to investigators’ assessment of objective tumor responses (RECIST v1.1 criteria) and according to each treatment arm. B. Swimmers’ plots of individual patients in each Arm 1

Figure 3a shows waterfall plots corresponding to the investigators’ assessment of best antitumor response (RECIST v1.1 criteria) from baseline to day 28 for the 30 evaluable patients. Figure 3b shows corresponding swimmer’s plots, including details about treatments they may have received following AMG 232 discontinuation. For example, patient 6-2005 was enrolled in Arm 2, cohort 3 (AMG 232 240 mg), received AMG 232 for only four days, and subsequently withdrew. Unfortunately, he died from metastatic melanoma 5.3 months after study enrollment.

**Fig. 4:**
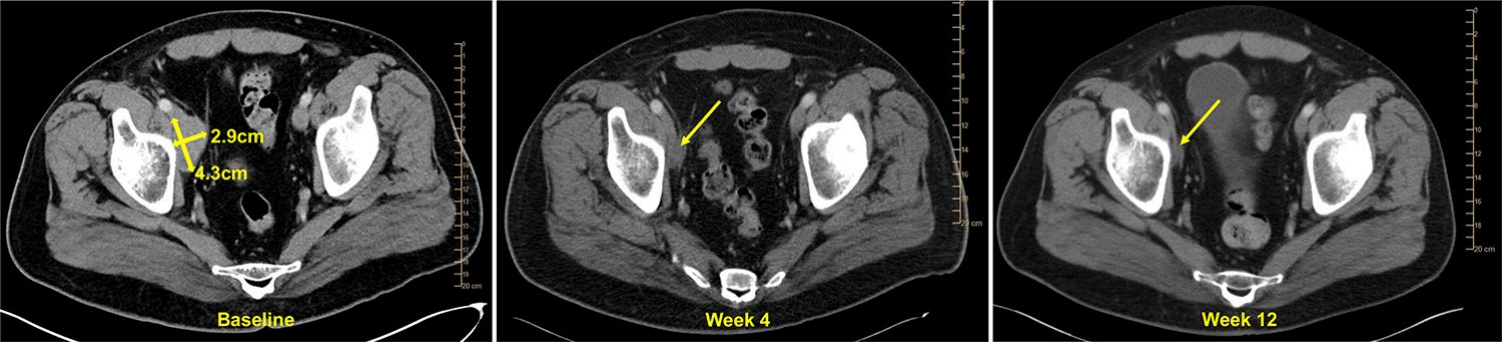
Complete antitumor response in the AMG 232, dabrafenib, trametinib study (Arm 1). Representative images were obtained from patient 6-1011’s CT scans with IV contrast at baseline, week 4, and week 8

In Arm 1 (n=10), the overall objective response rate was 80% (two complete and six partial responses) (Fig. 3a). Six out of eight responders did not have stage IV melanoma. Both Arm 1 complete responders (6-1012 and 6-1011) were treated with 180 mg of AMG 232, had oligometastatic, M1a, disease. Interestingly, patient 6-1011 received dabrafenib-trametinib-AMG 232 for nearly seven months; he subsequently chose to be observed. Figure 4 shows patient 6-1011’s representative images from IV contrast CT scans at baseline, week 4, and week 8 while on AMG 232, dabrafenib, and trametinib treatment. After >4 ½ years of follow-up, he did not have any melanoma recurrence. Two other Arm 1 patients (6-2007, 6-7007) stopped AMG 232 for toxicity and entered radiographic surveillance; they did not progress for at least seven months. In Arm 2 (n=20), three patients had a partial response, one in each dose cohort. The overall objective response rate was 15% (3/20, Fig. 3a). All three responders subsequently progressed within six months of study initiation. Interestingly, two of the three responders had M1c disease and *NRASQ61R* mutations. Five patients (25%) remained free of recurrence for at least five months.

Seven patients in Arm 1 were alive (70%) at the last follow-up (Fig. 3b). The median progression-free survival (PFS) was 19.0 months-not reached (range, 1.9 months-not reached), and the median overall survival (OS) for Arm 1 was 47.7 months (range, 2.3-62.3 months). Most responders in Arm 1 who developed toxicities from AMG 232 requiring discontinuation from the study elected to continue on BRAF/MEK inhibition and were alive at the last follow-up. Two of the three patients in Arm 1 who progressed (6-2007, 6-1008) had not previously received PD1/PD-L1 inhibitors; following progression from our study, they both received PD1 inhibitors and eventually progressed.

At the last follow-up, two patients in Arm 2 were alive (9.5%) (Fig. 3b). 18 patients died from MM and one patient died from pulmonary embolism not attributed to AMG 232. The median PFS (n=20, one non-evaluable) for Arm 2 was 2.8 months (0.9-14.1 months), and the median OS (n=21) was 8.7 months (range, 1.2-70-7 months). Four patients in Arm 2 survived for >2 years after study enrollment. Two of these patients had not previously received PD1 inhibitors. Patient 1-2002 (*NRAS*-mutant) developed a complete metabolic response to single-agent pembrolizumab and is free of disease. Patient 1-2009 (*NRASQ61R*-mutant) remained on single-agent pembrolizumab for an extended time while receiving radiation therapy to new lesions that would appear periodically. Patient 6-1010 (acral melanoma) had a single mutation (*NRASQ61K*) identified as part of the FoundationOne CDx assay following progression on AMG 232. He eventually became disease-free after a hip disarticulation surgery. The other two patients who had received PD1 inhibitors and ipilimumab prior to study enrollment (6-1001, 6-2002) developed antitumor responses to cytotoxic chemotherapy.

## Discussion

Our study showed that AMG 232 could be safely combined with trametinib alone or with concurrent dabrafenib-trametinib. The MTD was defined as 120 mg once daily for seven days of a 21-day cycle in both arms because DLTs occurred at the 180 mg and 240 mg dose cohorts. Interestingly, the MTD in our study was higher than that in a recently reported phase 1b study of the AMG 232-trametinib combination in relapsed/refractory acute myeloid leukemia [19]. Although gastrointestinal toxicities were seen in both the melanoma and leukemia studies, more serious and frequent hematologic toxicities occurred in the leukemia study, which may possibly account for the lower MTD established in the latter.

Preclinical data have shown that AMG 232 affects the p53 pathway, as measured by the upregulation of p53’s downstream effectors, p21, and the circulating macrophage inhibitor cytokine-1 (MIC-1), at doses as low as 5 mg/kg. However, the effect of AMG 232 on the p53 pathway was more consistent and pronounced at doses ranging between 25-100 mg/kg [16]. By this dose-effect relationship, an AMG 232 dose of 50 mg/kg was selected to test the efficacy of AMG 232 alone and in combination with dabrafenib plus trametinib in melanoma patient-derived xenograft models. This AMG 232 dose corresponds to a human daily dose of 250 mg [8]. Evidence that the biologically effective AMG 232 dose in humans is at least 240 mg daily comes from the phase I study of single-agent AMG 232 in solid tumors or multiple myeloma; in this study, significant increases in serum MIC-1 protein were only seen when the AMG 232 daily dose was higher than 240 mg [18]. Although we did not measure serum MIC-1 protein, or other relevant biomarker in our study, we can extrapolate from the biomarker results of the phase I solid tumor or multiple myeloma study to suggest that our recommended phase 2 dose of AMG 232 combined with trametinib alone or trametinib plus dabrafenib at 120 mg may not be sufficiently high to yield a biological effect that is significantly higher than the antitumor effect seen with MEK inhibition alone in patients with non-*BRAFV600*-mutant or with combined BRAF/MEK inhibition in patients with *BRAFV600*-mutant melanoma. In support of this notion, the response rate and PFS that we observed in patients from Arm 2 were not significantly different from that seen in the early studies of single-agent trametinib in non-*BRAFV600*-mutant MM [22], or in the more recent study of single-agent binimetinib in patients with *NRASQ61*-mutant melanoma [5]. Furthermore, we observed higher antitumor responses in Arm 1 with the highest dose of AMG 232 tested (180 mg) and perhaps a higher rate of stable disease in Arm 2 patients who received the highest dose of AMG 232 (240 mg). Nevertheless, we caution that the number of patients treated was too small to draw any definite conclusions. Also, we did not observe a significant effect of AMG 232 on the PK profiles of trametinib and dabrafenib, and vice versa, suggesting that there was no overall significant and clinically relevant PK interaction among the three drugs. Finally, the observed steady-state AMG 232 exposures (AUC after seven days of daily dosing) at the MTD of 120 mg in Arms 1 and 2 of this study were in general lower than corresponding exposures for the putative biologically effective dose of 240 mg AMG 232, as discussed above, in patients with solid tumors or multiple myeloma. We, therefore, conclude that we were unable to escalate AMG 232 to dose levels that could translate to substantial clinical activity in our study.

In summary, we have conducted a clinical study in MM that targets a protein, p53, that is not frequently mutated in MM and plays a fundamental role in melanoma’s decisions for cell death versus survival. The combination of AMG 232 with the standard of care dose of trametinib or trametinib plus dabrafenib was generally well tolerated, especially at relatively lower AMG 232 doses. However, the inability to escalate AMG 232 to higher doses underlies the vital role of p53 in human physiology [23], and perhaps may account for the lack of significantly higher clinical activity compared to trametinib alone or dabrafenib plus trametinib. Our experience of low activity due to the inability to escalate experimental drugs to biologically effective levels due to toxicity when combined with MAPK pathway inhibitors is similar to that seen with other promising targets in melanoma [24, 25]. 

## Supplementary Information

Below is the link to the electronic supplementary material.

Supplementary file1 (DOCX 25 KB)

## Data Availability

The datasets generated during and/or analysed during the current study are available from the corresponding author on reasonable request.

## Abbreviations

MM: metastatic melanoma
MAPK: mitogen-activated protein kinase
PD1: programmed cell death protein 1
PD-L1: programmed death-ligand 1
*MDM2*: the *murine double minute-2 proto-oncogene*
WT: wild-type
p53: the protein encoded by the *TP53* gene
PK: pharmacokinetics
MTD: maximum tolerated dose
NCI-CTCAE: the National Cancer Institute Common Terminology Criteria for Adverse Events
DLT: dose-limiting toxicity
AEs: adverse events
RECIST: Response Evaluation Criteria in Solid Tumors
ECOG: Eastern Cooperative Oncology Group
SAEs: serious adverse events
AUC_24hr_: area under the curve over 24 hours
C_max_: peak plasma concentration
t_max_: time to reach C_max_
t_1/2,z_: mean terminal half-life
CL/F: oral clearance
PFS: progression-free survival
OS: overall survival
MIC-1: macrophage inhibitory cytokine 1
