# Supplementary material for: Targeting wild-type TP53 using AMG 232 in combination with MAPK inhibition in Metastatic Melanoma; a phase 1 study
Source: Invest New Drugs. 2022 May 30;40(5):1051–65. doi: 10.1007/s10637-022-01253-3 (PMC9395504; doi:10.1007/s10637-022-01253-3)
Supplement: Supplementary file 1 — Supplementary file1 (DOCX 25 KB) [file 10637_2022_1253_MOESM1_ESM.docx]

**Supplementary Information**

**Patients and Methods**

*Inclusion and Exclusion Criteria*

Adults (≥18 years old) with a histologic or cytologic diagnosis of metastatic cutaneous or mucosal melanoma, adequate hematologic (hemoglobin ≥9 gm/L, absolute neutrophil count ≥1,500/μL, platelet count ≥100,000/μL), renal (serum creatinine <1.5 mg/dL or estimated glomerular filtration rate ≥50 mL/min/1.73m^2^), hepatic (aspartate aminotransferase [AST] and alanine aminotransferase [ALT] ≤2.5x upper limit of normal [ULN], alkaline phosphatase <2x ULN, total bilirubin ≤1.5x ULN), coagulation [PT/INR and PTT ≤1.5x ULN]), and cardiac function (left ventricular ejection fraction of at least the lower limit of normal) were enrolled. Additional eligibility criteria included measurable disease per response evaluation criteria in solid tumors (RECIST) version 1.1 guidelines and Eastern Cooperative Oncology Group (ECOG) performance status of 0-2.

Major exclusion criteria included prior treatment with BRAF and MEK inhibitors, unresolved toxicity from prior anticancer therapy, concurrent systemic therapy, or major surgery within 28 days of starting AMG 232. In addition, patients with brain metastases were excluded if they were not radiologically stable for six weeks or had completed brain radiation within six weeks from study entry. Other exclusion criteria included clinically significant bleeding, history of interstitial lung disease or pneumonitis, clinically significant cardiovascular risk (QTc interval ≥480 msecs, uncontrolled arrhythmias, treatment-refractory hypertension, class ≥II congestive heart failure, acute coronary syndrome), history of retinal vein occlusion, and use of potent CYP3A4 inhibitors.

*Study design*

This open-label multicenter study was designed to enroll subjects in 3 parts (**Figure 1**). Part 1 study design is described in the main text. Part 2 was designed as the dose expansion with both Arms planned to enroll patients according to doses and schedule previously established in Part 1 to confirm safety and tolerability and assess clinical activity (dose expansion). In Part 3, patients with *TP53WT*, *BRAFV600E/K*-mutant, and BRAF/MEK inhibitor-naïve metastatic melanoma would be randomized 1:1 to receive AMG 232 in combination with dabrafenib plus trametinib vs. trametinib plus dabrafenib alone (efficacy part).

In the event of toxicity, intermediate dose levels could be evaluated. In Arm 1, patients with *TP53WT* and *BRAFV600E/K* mutated melanoma received AMG 232 combined with trametinib plus dabrafenib. In Arm 2, patients with *TP53*/*BRAFV600E/K-wild type* melanoma received AMG 232 combined with trametinib alone. *BRAF* *non-V600* mutations were permitted (**Figure 1**). Enrollment across the two Arms was staggered, commencing initially in Arm 2. Only once the MTD of the AMG 232 combined with trametinib was defined in Arms 2 did the enrollment in Arm 1 with the triple combination of dabrafenib plus trametinib and AMG 232 commence.

*Safety and Response Assessment*

We defined the dose-limiting toxicity (DLT) period as the first 28 days of each dose-escalation cohort’s treatment cycle (DLT window). Fatigue, nausea, diarrhea, vomiting, neutropenia, thrombocytopenia, and lymphopenia did not meet DLT definition criteria directly. We defined DLTs as any grade ≥3 hematologic toxicity, including grade ≥3 febrile neutropenia, grade 4 non-febrile neutropenia persisting ≥4 days, grade ≥3 thrombocytopenia (with clinically significant bleeding), grade 4 thrombocytopenia, and grade ≥3 anemia attributable to AMG 232. Non-hematologic DLTs included grade ≥3 nausea, vomiting, or diarrhea enduring >48 hours despite standard medical management, grade 3 fatigue persisting >7 days or grade 4 fatigue, any grade ≥3 metabolic laboratory abnormalities that are not controlled within 48 hours by standard medical management. AST or ALT values ≥3x ULN plus serum total bilirubin level >2x ULN without signs of cholestasis and any other grade ≥3 AE attributable to AMG 232 were also considered DLTs. Irrespective of the underlying AE, failure to receive 85% of the schedule of AMG 232 during the DLT treatment period was deemed to reflect poor tolerability and was also considered a DLT.

*Pharmacokinetic Assessments*

Plasma concentrations of AMG 232 and its glucuronide were measured using a validated assay involving high-performance liquid chromatography followed by a tandem mass spectrometric (LC-MS/MS) detection method as described previously.^19^ Based on reported trametinib and dabrafenib half-life estimates (<https://www.novartis.us/sites/www.novartis.us/files/tafinlar.pdf>, <https://www.novartis.us/sites/www.novartis.us/files/mekinist.pdf>), we expected trametinib and dabrafenib levels to be at steady-state on cycle 1, day 21, and cycle 2, day 7. We, therefore, collected samples on these days to perform dabrafenib and trametinib PK analysis.

We determined plasma concentrations of trametinib, dabrafenib, and hydroxy- and desmethyl-dabrafenib metabolites with a validated assay using liquid-liquid extraction of samples spiked with the stable isotope internal standards for each analyte followed by analysis using LC-MS/MS detection. The validated concentration ranges for quantitation were 0.250 to 250 ng/mL for trametinib and 1.00 to 1,000 ng/mL for dabrafenib and its metabolites. We calculated concentrations using a linear regression calibration model with a 1/x^2^ weighting factor.

The following PK parameters were estimated: maximum observed plasma concentration (C_max_), time to reach C_max_ (t_max_), area-under-the-plasma concentration-time curve (AUC), terminal phase half-life (t_1/2,z_), and apparent total body clearance (CL/F). We performed noncompartmental analyses for PK parameter estimation using Phoenix® WinNonlin® version 6.4 or later software (Certara™, Princeton, NJ). To explore potential drug interactions among AMG 232, trametinib, and dabrafenib, we plotted mean (with standard deviation) concentration-time profiles of AMG 232, trametinib, and dabrafenib according to AMG 232 dose, arm, day, and cycle number. In addition, we conducted Wilcoxon matched-pairs signed-rank tests to compare AMG 232 C_max_ and AUC values of subjects with and without co-administration of trametinib and dabrafenib in Arm 1 and trametinib in Arm 2 and trametinib and dabrafenib C_max_ and AUC values of subjects with or without co-administration of AMG 232. We prepared descriptive statistics, tables, and graphs using Phoenix® WinNonlin® and Prism version 9.1.1 (GraphPad Software, San Diego, CA).

**Results**

*Pharmacokinetics*

Lower geometric mean AMG 232 exposures (C_max_ and AUC_24hr_) were observed across all dose cohorts when AMG 232 was combined with trametinib plus dabrafenib or trametinib alone following cycle 2, day 7, dosing compared to AMG 232 as a single agent following cycle 1, day 7, dosing. However, the effect of the combination of trametinib plus dabrafenib or trametinib alone on AMG 232 was not significant when comparing exposures (C_max_ and AUC_24hr_) following cycle 2, day 7, and cycle 1, day 7, dosing (p>0.05, Wilcoxon matched-pairs signed-rank tests for all dose cohorts in both arms except for the 240 mg dose cohort in Arm 2 where there was insufficient data to perform the test). However, AMG 232 exposures (C_max_ and AUC_24hr_) following cycle 1, day 7, dosing were not significantly different from the corresponding AMG 232 exposures following cycle 2, day 7, dosing for the 120 mg and 180 mg AMG 232 dose cohorts in either arm (Wilcoxon matched-pairs signed-rank tests, p > 0.05). Unfortunately, we could not test the comparison due to insufficient data for the 240 mg dose cohort.

**Figure 2B** shows plasma PK profiles of trametinib 2 mg PO QD at steady-state in combination with dabrafenib 150 mg PO BID or trametinib 2 mg PO QD alone at steady state with and without co-administration of AMG 232. **Table 4** shows pharmacokinetic parameter estimates of trametinib. Co-administration of AMG 232 with trametinib does not appear to affect trametinib PK. Trametinib was rapidly absorbed across dose cohorts with a median t_max_ of 2 hrs following cycle 1, day 21, dosing without AMG 232 co-administration and 1.0 to 3.0 hrs following cycle 2, day 7, dosing with AMG 232 co-administration. Trametinib exposures (C_max_ and AUC_24hr_) following cycle 1, day 21, dosing (without co-administration of AMG 232 at the end of the AMG 232 treatment-free period, Arm 1) were not significantly different from the corresponding trametinib exposures following cycle 2, day 7 (with co-administration of AMG 232, Arm 2) across the 120 mg and 180 mg dose cohorts (Wilcoxon matched-pairs signed-rank tests for all dose cohorts in both arms, p > 0.05). Unfortunately, we could not test the comparison due to insufficient data for the 180 mg dose cohort in Arm 2. In conclusion, our study has observed PK parameter estimates of trametinib 2 mg QD consistent with those reported previously following repeat-dosing of trametinib monotherapy.^21^

**Figure 2C** shows plasma PK profiles of dabrafenib 150 mg PO BID at steady-state combined with trametinib 2 mg PO QD with and without co-administration of AMG 232 in Arm 1. **Table 5** shows PK parameter estimates of dabrafenib. Dabrafenib exposures were not significantly altered when dabrafenib and trametinib were combined with and without AMG 232. Dabrafenib was rapidly absorbed with a median t_max_ of 1 hour following cycle 1, day 21, dosing without AMG 232 co-administration and 2.0 hours following cycle 2, day 7, dosing with AMG 232 co-administration. Geometric mean dabrafenib exposures (C_max_ and AUC_6hr_) were lower when dabrafenib and trametinib were co-administered with AMG 232 than without AMG 232. However, significant differences in exposures were not observed (p > 0.05 for 120 mg AMG 232 dose cohort, Wilcoxon matched-pairs signed-rank test for the 120 mg AMG 232 dose cohort p > 0.05; significance testing not performed for 180 mg AMG 232 dose cohort due to insufficient data). Thus, PK parameter estimates for dabrafenib 150 mg BID in this study were within the range of those observed in previous studies following repeat-dosing of dabrafenib monotherapy (<https://www.accessdata.fda.gov/drugsatfda_docs/nda/2013/202806orig1s000clinpharmr.pdf>).
